# Deoxygenation impacts on Baltic Sea cod: Dramatic declines in ecosystem services of an iconic keystone predator

**DOI:** 10.1007/s13280-021-01572-4

**Published:** 2021-06-01

**Authors:** Alessandro Orio, Yvette Heimbrand, Karin Limburg

**Affiliations:** 1 Department of Aquatic Resources, Institute of Marine Research, Swedish University of Agricultural Sciences, Turistgatan 5, 453 30 Lysekil, Sweden; 2 Department of Aquatic Resources, Institute of Coastal Research, Swedish University of Agricultural Sciences, Skolgatan 6, 742 42 Öregrund, Sweden; 3 College of Environmental Science and Forestry, State University of New York, Syracuse, NY USA

**Keywords:** Baltic Sea, Cod (*Gadus morhua*), Deoxygenation, Ecosystem services, Hypoxia, Maximum length

## Abstract

The intensified expansion of the Baltic Sea’s hypoxic zone has been proposed as one reason for the current poor status of cod (*Gadus morhua*) in the Baltic Sea, with repercussions throughout the food web and on ecosystem services. We examined the links between increased hypoxic areas and the decline in maximum length of Baltic cod, a demographic proxy for services generation. We analysed the effect of different predictors on maximum length of Baltic cod during 1978–2014 using a generalized additive model. The extent of minimally suitable areas for cod (oxygen concentration ≥ 1 ml l^−1^) is the most important predictor of decreased cod maximum length. We also show, with simulations, the potential for Baltic cod to increase its maximum length if hypoxic areal extent is reduced to levels comparable to the beginning of the 1990s. We discuss our findings in relation to ecosystem services affected by the decrease of cod maximum length.

## Introduction

Ecosystem services are defined as ‘‘the benefits people obtain from ecosystems’’ (Millennium Ecosystem Assessment 2005) and are mainly divided into provisioning, regulating and maintenance, and cultural services (Haines-Young and Potschin 2013). The economic aspect of many ecosystem services is straightforward and well known to the public, whereas the underlying ecological aspects are not as obvious. For example, the ecosystem service provided by fish as a source of food has a monetary value in form of market price, while the value of fish as an important part of a functioning aquatic food web is not easily quantifiable in economic terms (Holmlund and Hammer 1999; Limburg 2009). Aquatic ecosystem services generally are products of diverse, fully functioning ecosystems with many connections, e.g. (Hillman et al. 2018) and good environmental quality. Water quality status includes a number of variables, with good oxygen conditions being a paramount feature.

Decreased levels of dissolved oxygen (hypoxia and anoxia) in global oceans and coastal zones is a growing problem around the world generated primarily by eutrophication and anthropogenic emissions of greenhouse gases (Diaz and Rosenberg 2008; Breitburg et al. 2018). Deoxygenation is now considered a global problem (Laffoley and Baxter 2019). Hypoxic waters can affect organisms through direct mortality, alteration of metabolism and growth, forced migration, habitat contraction, increased susceptibility to predation, or changes in prey availability (Rabalais et al. 2001, 2002; Breitburg 2002; Diaz and Rosenberg 2011; Hinrichsen et al. 2011; Levin 2018). In the case of mobile organisms, such as fish, the effects of hypoxic water are mostly indirect and connected to habitat contraction and changes in spatial distribution (Craig and Crowder 2005; Eby et al. 2005; Bijma et al. 2013; Chu and Tunnicliffe 2015). However, there are different mechanisms that link fish growth to oxygen concentrations as, for example, physiological stress due to exposure to hypoxia increasing metabolic costs, or overcrowding in normoxic areas resulting in density-dependent reduction of growth rates from resource depletion or interference competition (Breitburg 2002; Eby et al. 2005; Pollock et al. 2007).

The Baltic Sea contains the largest anthropogenic hypoxic area in the world (Carstensen and Conley 2019). It has been driven to this state mainly by nutrient loading producing eutrophication, but also climate change, warming the seawater (Carstensen et al. 2014a, b). The solubility of oxygen in water decreases with increased temperature and, in the Baltic Sea, monitoring results have shown an increase in annual mean sea-surface temperature of around 1 °C per decade from 1990 to 2008 (HELCOM 2013). In addition, warmer water temperature also increases the decomposition rate of organic matter, further exacerbating the hypoxia (Carstensen et al. 2014a).

**Fig. 1:**
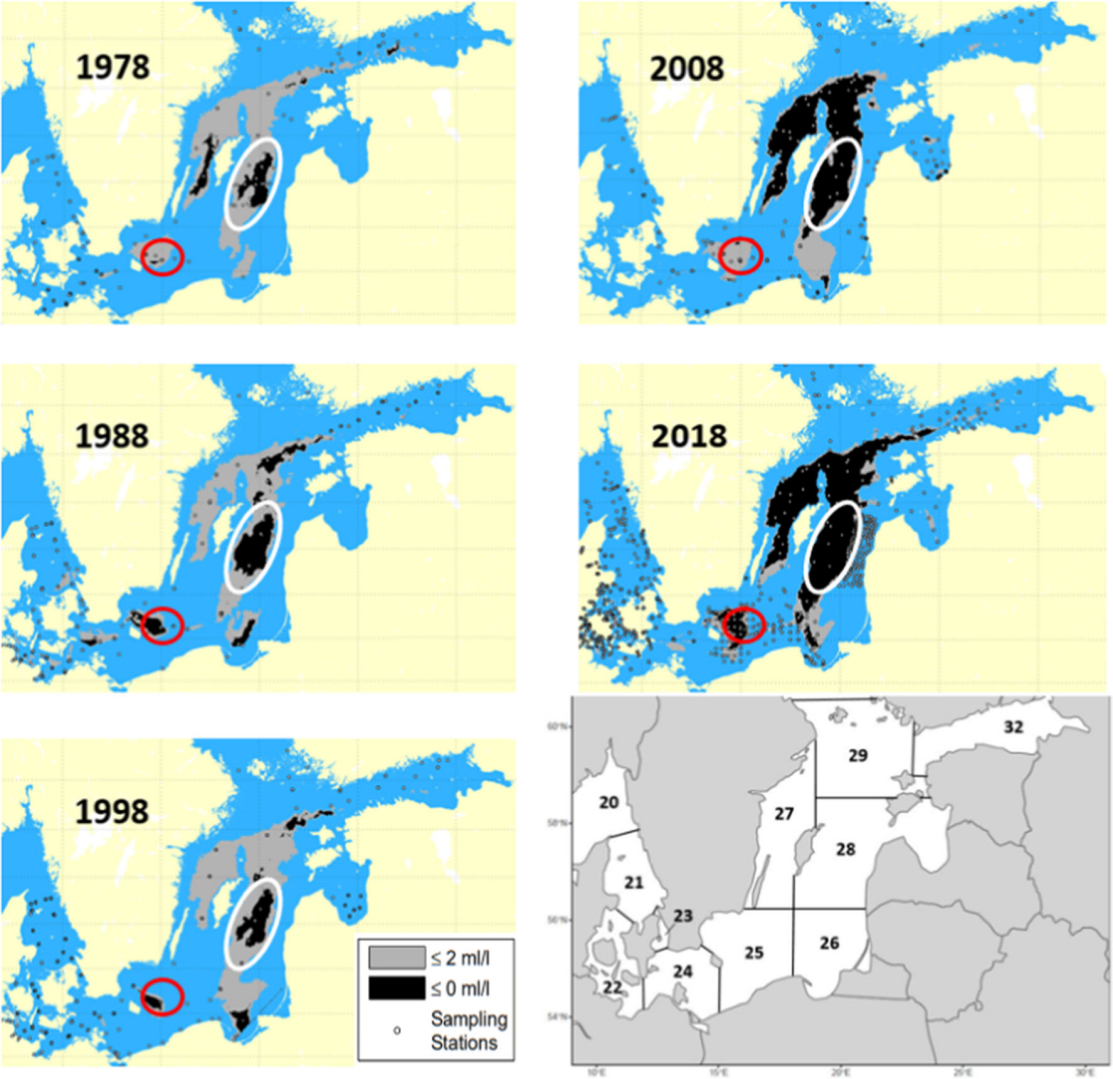
Changes in the extent of hypoxic and anoxic bottom water in autumn in the Baltic Sea during the years 1978, 1988, 1998, 2008 and 2018, reported from the annual oxygen survey performed by the Swedish Meteorological and Hydrological Institute. The areas in grey correspond to areas with oxygen levels below 2 ml l^−1^; black areas are anoxic, totally depleted of oxygen. Gotland Deep (white oval) and Bornholm Deep (red circle) are historic spawning and nursery areas for Baltic cod. The bottom right panel shows the ICES Sub-Divisions for the same area (Hansson and Andersson 2013; Hansson et al. 2009, 2018)

The Baltic Sea is a semi-enclosed, shallow, brackish inland sea in northern Europe (Table 1). The Baltic Sea is non-tidal with a large drainage area (watershed area: sea surface area = 4.17) and connects to the North Sea via narrow straits acting as thresholds between the brackish and marine systems. Outflow conditions usually dominate the water exchanges with the North Sea. In recent years, major North Sea inflows, sufficiently large to ventilate the oxygen-poor bottom layers, occur only once per decade compared to five to seven per decade during the twentieth century (Mohrholz et al. 2015). Because of this stagnation, hypoxic areas have expanded from 5 000 to 60 000 km^2^ over the past century, increasing fivefold during the last two decades (Hansson and Andersson 2013; Carstensen et al. 2014a). Bottom water hypoxia is a key factor shaping the benthic community in the Baltic, leading to extirpations of macrofaunal biomass on the sea bottom; with repercussions on all trophic levels of the Baltic Sea ecosystem (Karlson et al. 2002). The areas that are especially affected by hypoxia and anoxia are the deep, southern areas, the Gotland Deep and Bornholm Deep (Furman et al. 2014) (Fig. 1).

**Table 1:** Characteristics of the Baltic Sea

Surface area	420 000 km^2^	HELCOM (2018)
Drainage area	1.7 × 106 km^2^	HELCOM (2018)
Volume	21 700 km^3^	BACC Author Team (2008)
Average water residence time	30 years	Korpinen et al. (2010)
Mean depth	54 m	Furman et al. (2014)
Maximum depth	459 m	Furman et al. (2014)
Salinity	0–30 PSU	HELCOM (2018)

We focus here on the Baltic cod (*Gadus morhua*) as a producer of provisioning, regulating and cultural ecosystem services (Haines-Young and Potschin 2013), and how these are impacted by deoxygenation. Cod is an economically and ecologically key fish species in the Baltic Sea. In the late 1970s, the favourable water conditions for cod spawning, high abundance of food for cod larvae, and a decrease in fishing mortality produced a “cod boom” in the 1980s with enormous landings (around 400 thousand tons per year; Köster et al. 2005; Casini 2013; ICES 2020). The subsequent period, characterized by overfishing in combination with worsening water quality conditions and hypoxia intensification, caused the collapse of the stock. At the same time, hypoxia-induced habitat compression led to crowding and density-dependent effects (Casini et al. 2016). Other hypotheses have been suggested to explain the collapse of the Eastern Baltic stock such as the limitation in prey availability (Eero et al. 2012) and an increase in seal predation on cod that also transmitted parasites (Mehrdana et al. 2014; Nadolna and Podolska 2014).

Over the past 40 years, the hypoxic and anoxic regions correspond, to a large extent, to previously known spawning areas for the Eastern Baltic cod stock (Cardinale and Svedäng 2011). The stock’s reproduction, in fact, is restricted to regions where salinity exceeds 11 PSU, which is the limit for sufficient buoyancy for fertilized cod eggs, and oxygen concentration > 2 ml l^−1^ (Nissling and Westin 1997; Hinrichsen et al. 2017). Highest salinity waters are dense and stratify to the bottom in the deeper areas of the Baltic, and therefore, are often hypoxic (Nissling and Westin 1997). Furthermore, the metabolic performance of cod is reduced by hypoxia, which causes a decrease in energy available for growth, swimming and feeding activities (Chabot and Claireaux 2008; Claireaux and Chabot 2016). In addition to all these effects, the oxygen deficiency at the bottom also decreases the production of benthic fauna that cod feed upon (Casini et al. 2016; Neuenfeldt et al. 2020).

In recent years, the Eastern Baltic cod have decreased in average maximum size, from around 80 cm in the 1980s to around 40 cm today (Orio et al. 2017), with a similar halving of body condition (a fatness index) over the same period (Casini et al. 2016; Svedäng and Hornborg 2017). Moreover, in the same period there have been signals of a decrease in cod growth (Svedäng and Hornborg 2014; Eero et al. 2015). However, due to unreliable age data it is difficult to draw a conclusion concerning the causes of the changes in size (ICES 2014; Eero et al. 2015). These changes, together with the loss of large cod (particularly females), have been proposed as some of the causes of an ecological regime shift that affected the Baltic Sea around the late 1980s (Gårdmark et al. 2015) producing trophic cascades (Casini et al. 2009), which affected the entire ecosystem and in turn the ecosystem services provided.

In this paper, we highlight the importance of large cod in the production of ecosystem services in the Baltic. Large fish are highly appreciated by people, inspiring admiration and awe, as evidenced for example by the popular show “River Monsters” (Wade 2011). Our aim is to explore the utility of this demographic property in terms of generating ecosystem services. We approach this by analysing the changes in the maximum size of Baltic cod to test various factors that might explain changes in cod size over recent decades. We first apply a generalized additive model (GAM) to investigate the links between the loss of large cod, food availability, fishing mortality and deoxygenation in the Baltic Sea. In particular, focusing on the effect of deoxygenation, we then simulate different oxygen scenarios and investigate the potential future change of maximum length of cod. Finally, we identify ecosystem services linked to the decrease in maximum length of Baltic cod focusing on the economic, ecological, and societal perspectives.

## Materials and methods

### Available data

**Fig. 2:**
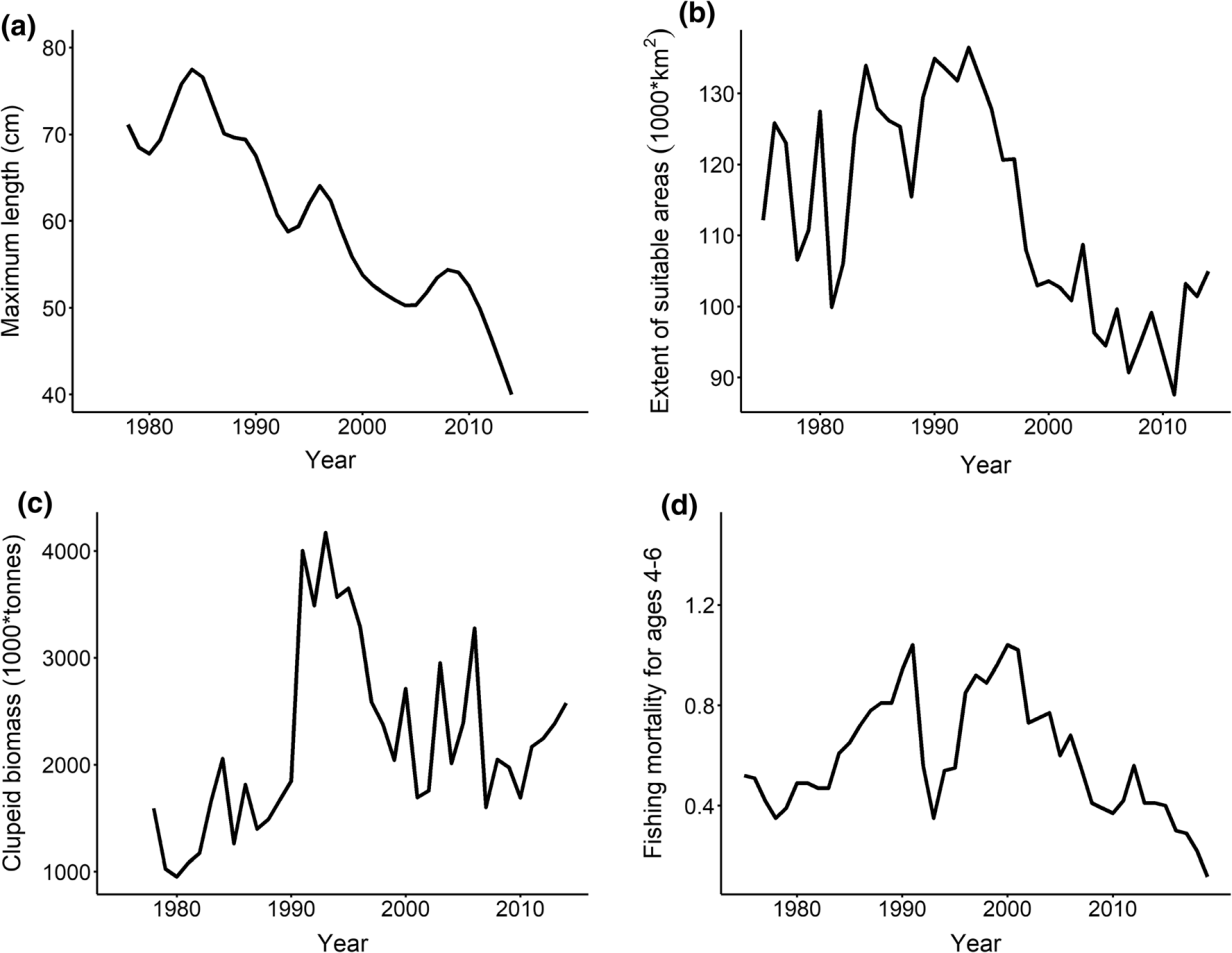
Time-series of **a** average maximum length of cod, **b** extent of suitable areas (oxygen concentration ≥ 1 ml l^−1^), **c** clupeid biomass (herring + sprat), **d** fishing mortality. See text for data sources

The time-series of average maximum length (*L*_max_) (Fig. 2a) of cod in sampling Sub-Divisions (SDs) 25–28 (Fig. 1) from 1978 to 2014 was retrieved from Orio et al. (2017) in which they modelled *L*_max_ of cod caught in historical and modern trawl surveys using the Generalized Additive Model approach (GAM; Hastie and Tibshirani 1990).

Following Casini et al. (2016), we chose a dissolved oxygen concentration > 1 ml l^−1^ as a threshold for minimum habitat suitability for adult Baltic cod. This threshold for hypoxia is justified because Baltic cod has been shown to avoid oxygen concentrations below this level (Schaber et al. 2012). The time-series of the extent of suitable areas, as well as clupeid biomass (Fig. 2b, c) were retrieved from Casini et al. (2016). We examined the extent of suitable areas for cod (km^2^) within the 20–100 m depth interval from 1975 to 2014. The time-series of clupeid biomass represents availability of the main pelagic prey of cod (herring and sprat), the ages 1–8, in SDs 25–28 from 1978 to 2014. These data were collected during the autumn Baltic International Acoustic Survey (BIAS) (ICES 2018), and historical acoustic surveys from the Department of Aquatic Resources, Swedish University of Agricultural Sciences.

The time-series of fishing mortality (Fig. 2d) was retrieved from the last accepted assessment of the Eastern Baltic cod stock (ICES 2020) and consists in the fishing mortality of cod for the ages 4–6 from 1975 to 2019.

### Generalized additive model for *L*_max_

To analyse the effect of the different predictors on cod *L*_max_ between 1978 to 2014, we performed a GAM using a Gaussian distribution since the *L*_max_ values were normally distributed (Hastie and Tibshirani 1990). All variables were expressed as standardized anomalies prior to analysis (*X′* = *X* – mean/standard deviation). The full model was formulated as follows:

$$L_{\max } = \beta + f_{1} \left( {{\text{suitable}}\,{\text{areas}}} \right) + f_{2} \left( {{\text{clupeid}}\,{\text{biomass}}} \right) \, + f_{3} \left( {{\text{fishing}}\,{\text{mortality}}} \right) \, + \varepsilon$$

where *β* is an overall intercept, *f*_*i*_ are natural cubic splines and *ε* is an error term. Model selection was done through a backward stepwise selection approach based on statistical significance (Wood 2006). From the full model, the non-significant predictor with the lowest significance level was excluded at each step and the model run again. This procedure was repeated until all the predictors were significant (final model). To obtain ecologically significant models and to avoid overfitting, we set a limit to the maximum degrees of freedom (number of knots, *k*) allowed to the smoothing functions of the explanatory variables (*k* = 4).

To quantify the importance of the extent of suitable areas for cod on the changes of *L*_max_ we performed two different tests. First, we removed the variable concerning the extent of suitable areas from the final model and checked the difference in deviance explained between the two models. Second, we ran a model using only the extent of suitable areas for cod as explanatory variable and checked again the amount of deviance explained by the model.

### Simulation of changes in *L*_max_ based on different oxygen scenarios

To analyse the potential effects of changes in the extent of suitable areas for cod on *L*_max_ in the future, we performed a simulation exercise. We used the final model to predict the increase or decrease of *L*_max_ using four different oxygenation scenarios. In all the different scenarios we set the clupeid biomass as the average of the time-series (2207 thousand tonnes). The four scenarios differed in the extent of suitable area for cod:

1. Minimum extent of suitable area (87 thousand km^2^ in 2011)
2. Maximum extent of suitable area (136 thousand km^2^ in 1993)
3. Minimum extent of suitable area reduced by 2 standard deviations (58 thousand km^2^)
4. All areas become suitable (146 thousand km^2^)

## Results

### Generalized additive model for *L*_max_

All the explanatory variables with the exception of fishing mortality were retained in the final model for *L*_max_ because they were significant (*p* < 0.05). Summary statistics of the final model as well as the model without the extent of suitable areas for cod and the model with only the extent of suitable areas for cod are presented in Table 2.

**Table 2:** Summary statistics of the GAMs used to model the changes of *L*_max_ of cod. df = degrees of freedom; Dev% = explained deviance

Model	df	Variables retained	Dev%
Final model	3.6	Extent of suitable areas for cod, Clupeid biomass	72.0
Final – extent of suitable areas	3.2	Clupeid biomass	35.8
Only extent of suitable areas	3.7	Extent of suitable areas for cod	48.6

**Fig. 3:**
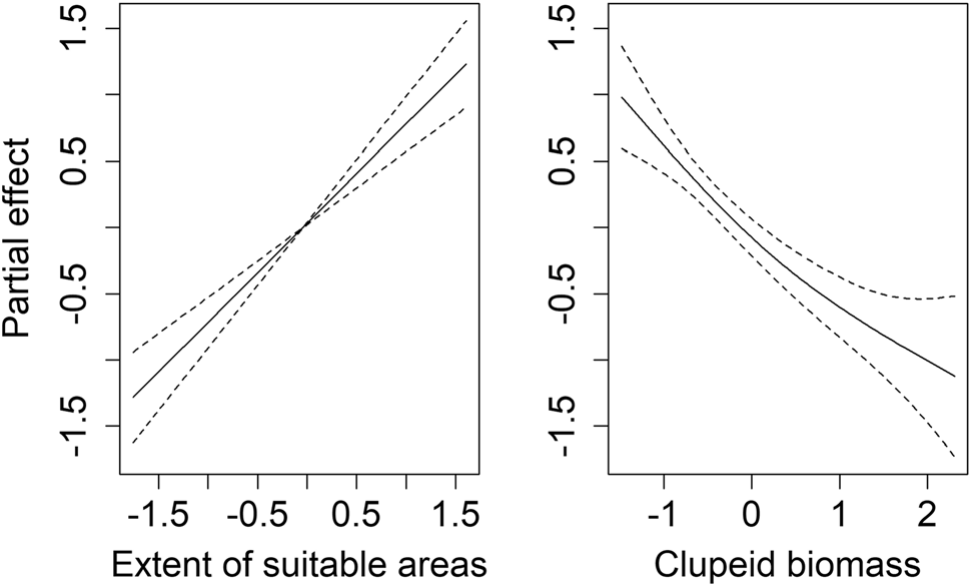
Results of the final model for *L*_max_. The partial effects of each predictor are shown (solid lines) with the associated confidence intervals (dashed lines). Values on the *x* axis represent standardized anomalies while values on the *y* axis are centred for each smooth term

The final model explained 72% of the deviance. Removing the predictor of the extent of suitable areas for cod resulted in a change in deviance explained of − 36.2%. In contrast, the model including only the extent of suitable areas for cod explained 48.6% of the deviance. Visual inspection of the residuals did not reveal any major departure from the model assumptions. The partial effects of the final model are presented in Fig. 3. The partial effect of the extent of suitable areas for cod showed a positive linear effect on the cod *L*_max_ while the clupeid biomass showed a negative one.

### Simulation of changes in *L*_max_ based on different oxygen scenarios

**Fig. 4:**
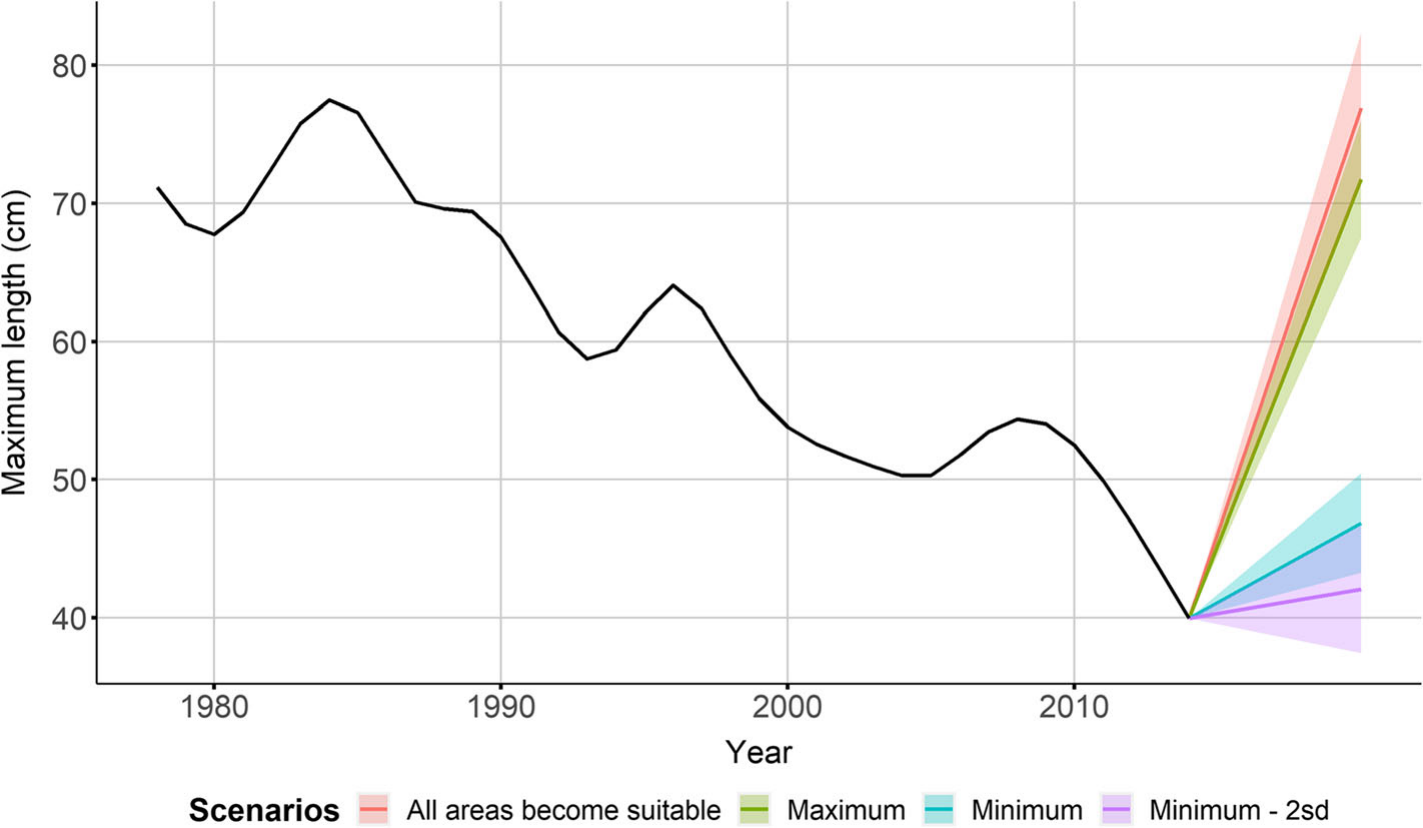
Results of the simulation exercise on the changes of *L*_max_ of cod based on different oxygen scenarios. The coloured bands represent the 95% confidence intervals. Note that the predictions of the different scenarios do not refer to a specific year, but they are just plotted in the future

The results of the simulation exercise are presented in Fig. 4. Simulated average *L*_max_ of cod was predicted to increase to levels similar to the mid-1980s (between 70 and 80 cm) if the extent of suitable areas is increased to the level of 1993 (maximum extent scenario) or if there were no areas between 20 and 100 m depth with oxygen concentration < 1 ml l^−1^. On the contrary, if the anoxic and hypoxic areas were to increase (the scenarios of minimum extent and minimum extent – 2 standard deviations) the average *L*_max_ of cod was predicted to increase slightly with values between 40 and 50 cm.

## Discussion

### Modelling the decline in maximum length of cod

In this study, we attempt to link the increase in hypoxic areas with the decrease in maximum length of Baltic cod. Our model results show that the extent of suitable areas for cod have substantial explanatory power in predicting the changes in the maximum length of cod. In particular, when modelling the changes in *L*_max_ using only our oxygen-related variable, the effect of the extent of suitable areas explains almost half of the deviance in our data. Furthermore, the link between oxygen and *L*_max_ appears to be linear and positive, so that an increase in bottom oxygen conditions is reflected in an increase of the length of cod. However, it is important to remember that in our model, by not lagging the effects of anoxic areas on the changes in maximum length, we are assuming a direct effect of oxygen concentration on the presence of large individuals, i.e. an effect on mortality. This could be correct in case of an increased vulnerability of large cod, for example, to fishing, due to hypoxia-induced habitat loss and subsequent concentration of individual in areas subject to higher fishing pressure. Therefore, in the future it will be interesting to test the effect of anoxic areas by lagging the extent of suitable areas a number of years in order to test the indirect effects of oxygen on growth rates since it has been shown in literature that exposure to low oxygen concentrations causes reduced growth in fishes (Breitburg 2002 and references therein). In fact, in a recent study that used a geochemical proxy in fish otoliths for hypoxia exposure, Limburg and Casini (2018) found that cod most exposed to hypoxia (75–100% per year) had 39% less growth by Age-3, and 64% lower weight, than cod least exposed (0–25% per year).

The effect of clupeid biomass on cod *L*_max_ showed in our model is almost linear and negative. This explanatory variable was added in the model at first as an index of prey abundance for cod. However, the negative effect reported by the model suggests that this variable actually indicates a regime shift, rather than prey availability per se The Baltic Sea experienced a regime shift in the mid-1980s and moved from a cod-dominated ecosystem to a clupeid-dominated one, characterized particularly by the high increase in sprat biomass (Casini et al. 2009; Gårdmark et al. 2015). The cod-dominated regime (i.e. before the 1990s) was characterized by high cod *L*_max_ and stock size which could control the size of the clupeid stocks via predation, keeping them at a lower level compared to the clupeid-dominated regime. After the regime shift the clupeid stocks experienced a predation release and increased their abundance, especially sprat. In this new regime cod *L*_max_ has also declined. Therefore, a low biomass of clupeid appears, counterintuitively, to have a positive effect on cod *L*_max_. To strengthen this hypothesis, we ran our models using sprat biomass, instead of clupeid biomass (i.e. sprat and herring), as a stronger indicator of the Baltic regime shift. The results of the model do not change significantly, lending support to our proposed explanation.

Our final generalized additive model did not include fishing mortality as an explanatory variable. This is notable, as a large literature exists on the impacts of fishing on size reductions in fishes (see reviews in Kuparinen and Merilä 2007; Fenberg and Roy 2008; Sharpe and Hendry 2009), including leading to evolutionary changes (Therkildsen et al. 2019). Indeed, separating the effects of fishing from climate change impacts is often difficult or impossible to do. Our analysis may present a clear example where a climate-driven effect (deoxygenation) impairs fish size without the confounding effect of fishing mortality, and may support other projections based upon metabolic theory.

The simulation exercise we conducted predicted different trends in maximum length based on different scenarios of oxygen levels in the Baltic Sea. We note that these models are not strictly mechanistic, but rather, project changes in a phenomenological (correlative) manner. The results of the simulation show the potential for cod in the Baltic to increase its maximum length in the future given an average abundance of clupeid biomass and a reduction of hypoxic areas to levels comparable to the beginning of the 1990s. However, the scenarios with increasing hypoxic areas show a slight increase of the maximum length of cod. This slight increase is due to the fact that the average clupeid biomass used in the predictions is lower than that of the final year in our model, resulting in an overall positive effect on cod *L*_max_ despite the increase in hypoxic areas (see above for the discussion on the clupeid biomass effect). This is also supported by the fact that predictions from the model including only extent of suitable areas show a decrease in maximum length of cod with a decrease in extent of suitable areas. A recent report from the Baltic Sea Oxygen Survey shows that in 2019, 33% of the total bottom area was affected by anoxia and hypoxia compared to 18% during the period of 2014–2016 (Hansson et al. 2019). Although we cannot directly validate our predicted trends of maximum length, we can still conclude that, since the expansion of hypoxia continues in the Baltic Sea, the scenarios showing a stable trend or a slight increase in maximum length connected with an increase extent of hypoxic areas are the most plausible given also an increase in clupeid biomass.

### Consequences for ecosystem services associated with large cod

The observed changes in *L*_max_, and the importance of bottom oxygen concentration in explaining them, have severe implications for many ecosystem services provided by the Baltic Sea. To analyse those, in Table 3 we have listed ecosystem services that are directly affected by the decrease in maximum length of the Baltic cod and the related consequences.

**Table 3:** Ecosystem services that are directly affected by the decrease in maximum length of the Baltic cod and related consequences

Ecosystem service	Implication	References
Reproduction potential	Older, larger female fish produce more young per year and often exponentially more than younger females	Hixon et al. (2014) Vallin et al. (1999)
	The larvae of older females may be larger, with greater fat reserves that can aid growth and survival	Mion et al. (2018)
	The potential fecundity for Baltic cod is mainly positively related to fish length, but body condition factor and hepatosomatic index also contributed significantly to explain the variation in potential fecundity	Barneche et al. (2018)
	Larger mothers reproduce disproportionately more than smaller mothers in not only fecundity but also total reproductive energy	Vallin and Nissling (2000)
	Large females are found to produce larger eggs with neutral egg buoyancy at a lower salinity, implying egg development in more favourable oxygen conditions	Vallin and Nissling (2000)
A top-down controlled balanced ecosystem	Trophic cascades	Casini et al. (2009)
	Regime shifts Size- and life-stage-dependent processes of predators and prey	Gårdmark et al. (2015)
	The loss of large predators, such as cod, causes an increased abundance of planktivorous fish that suppress the zooplankton population with resulting shifts in phytoplankton composition and abundance, reduced water transparency, more frequent algae blooms, increased hypoxia and reduced reproduction potential	Casini et al. (2008)
Size and biomass	Decreased individual growth and truncated size structures affect population biomass	Svedäng and Hornborg (2017)
The Eastern Baltic cod population as a sustainable natural resource	Socio-economic effects Large sized cod has a higher market value than small sized cod, so reduction in size leads to reduced income for the commercial fishery	Svedäng and Hornborg (2015)
Sportfish density for recreational fishery	The possibility to catch large cod attracts recreational fishers, which increases income from tourism in coastal communities. However, it could also affect the total catch in the form of unreported landed cod	Strehlow et al. (2012)

**Fig. 5:**
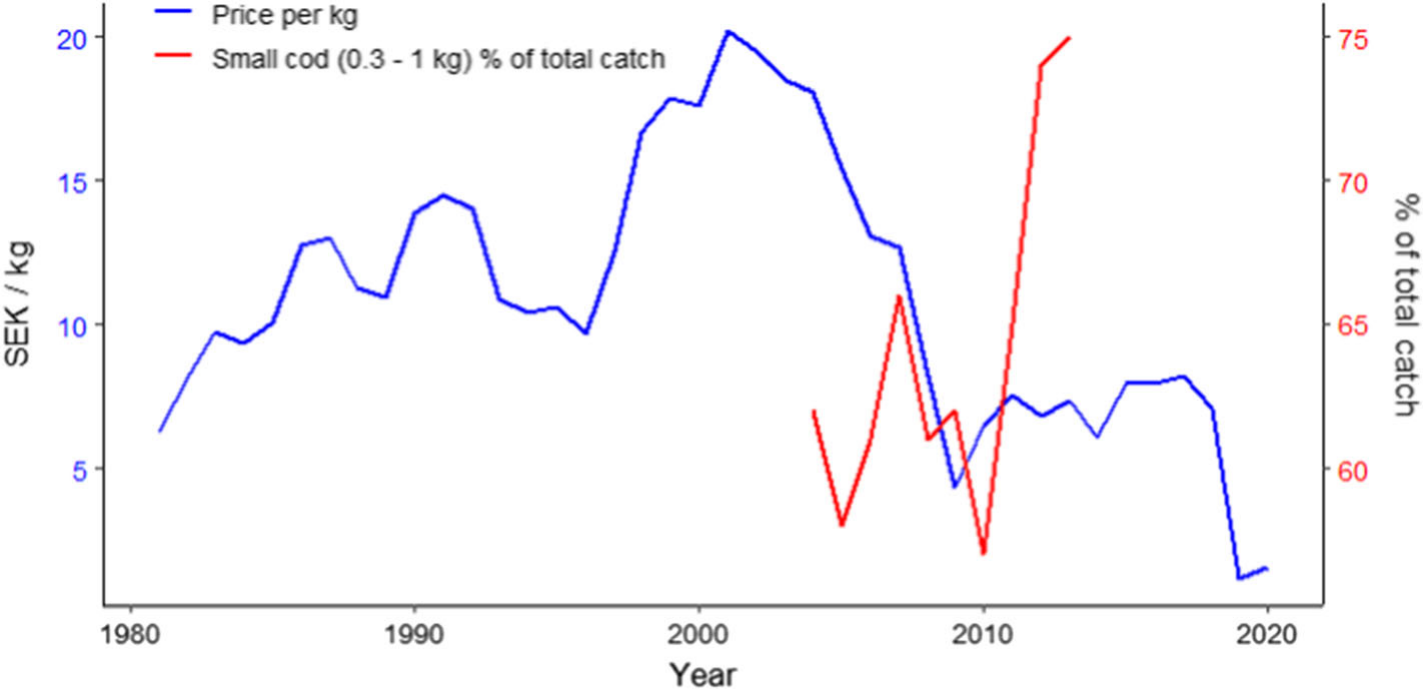
Time series of the value of Swedish Eastern Baltic cod catches, normalized by the weight of the catch and adjusted to 2020 Swedish kronor (SEK). Superimposed is the percentage of smallest-sized cod in the commercial catches as reported by Davelid et al. (2014). Note the approximate inverse relationship during the overlapping time period. Values of cod catches from 1981 to 1998 were extracted from the Statistical Yearbooks of Sweden (Statistics Sweden 1983–2000) and 1999–2020 from the Swedish Agency for Marine and Water Management (2020). All values were inflation-adjusted according to Statistics Sweden (https://www.scb.se/hitta-statistik/sverige-i-siffror/prisomraknaren/)

Some of the ecosystem services identified in Table 3 have a potential for serious economic consequences, many of which have likely already occurred. The ecosystem service that is most easily identifiable as having socio-economic impact caused by a decrease in cod *L*_max_ is decreased cod stock biomass. A stunted size distribution affects the cod stock biomass negatively (Eero et al. 2012; Svedäng and Hornborg 2017), which in turn affects fishing opportunities by reducing the fishable biomass and subsequent revenue. Davelid et al. (2014) found that landings of Class 1 cod (> 7 kg) from the Baltic declined nearly sevenfold between 2004 and 2013, while landings of Class 5 cod (0.3–1 kg) increased 1.2-fold. Prices dropped by 94% from 2001 to 2019 for cod landed in the Eastern Baltic Sea (Fig. 5). Processing cod fillets has become less economical, as it takes more time to fillet the scrawnier cod, and the meat is of poorer quality (Svedäng and Hornborg 2014). In addition to that, the largest cod also represent the most valuable size class on the market (Svedäng and Hornborg 2015), so loss of large cod reduces fishing revenue even more.

Recreational fishing experiences also constitute an ecosystem service that is negatively affected by the reduction of cod *L*_max_. The sportfish density and the possibility to catch trophy-sized cod attract recreational fishers to the Baltic Sea, which leads to increased income from tourism in the coastal communities. Therefore, the disappearance of large cod from the Baltic will have, and probably already had, economic impacts in terms of the economy of the coastal communities that work with recreational fishers. However, it is important to remember that, since the landings from recreational fishers are not yet well integrated into stock assessments, an increase or decrease in the amount of cod landed by recreational fishers has an impact on the uncertainties regarding the fishing mortality estimated by the assessment (Strehlow et al. 2012).

The Eastern Baltic cod population as a sustainable natural resource is an ecosystem service that has been taken for granted in the past, but today research projects, education and media contribute to raising public awareness of the stock’s threatened state. An example of ecosystem service related to the wellbeing of the cod stock and threatened by the decrease in cod maximum length is the reproductive potential of cod. In terms of potential fecundity, this is positively related to fish length (Barneche et al. 2018; Mion et al. 2018). Old, large females can produce a higher quantity of eggs of better quality than smaller-sized females (Hixon et al. 2014). Due to the enhanced quality in terms of nutrients and essential fatty acids, hatched-out larvae are larger and with greater energy reserves, improving the odds for rapid growth and survival (Vallin et al. 1999; Hixon et al. 2014). These factors imply that the loss of large cod individuals has the potential of reinforcing the current poor status of the Eastern Baltic cod by reducing its reproductive potential, creating a feedback loop that continues to produce smaller cod with poor recruitment. Furthermore, older, larger fish of many species including cod become better at finding the right places to spawn, learning with experience and increasing population resilience (Rose 1993; Secor 2000).

Another implication of the loss of large cod individuals on Baltic Sea ecosystem services can be found when analysing some of the reasons behind the regime shift that happened in the late 1980s in the Baltic Sea. In fact, size- and life-stage dependent processes between predator and prey can, when unbalanced, cause regime shifts and trophic cascades (Casini et al. 2009; Gårdmark et al. 2015). The loss of large top predators reduces a regulation service, i.e. it decreases the predation on planktivorous fish, which in turn alters the dynamics of zooplankton and phytoplankton. For the Eastern Baltic cod and the Baltic Sea’s top-down controlled balanced ecosystem, this means increased abundance of clupeids that graze down the zooplankton community, which in turn favours phytoplankton growth. Phytoplankton blooms in recent decades have been characterized by widespread summertime blooms of cyanobacteria able to capitalize on available phosphorus and N fixation (Kahru and Elmgren 2014). Increased phytoplankton production can then lead to reduced water transparency (Fleming-Lehtinen and Laamanen 2012), increased loading of dead organic matter, and consequent oxygen drawdown as the dead plankton cells are decomposed (Middelburg and Meysman 2007).

**Fig. 6:**
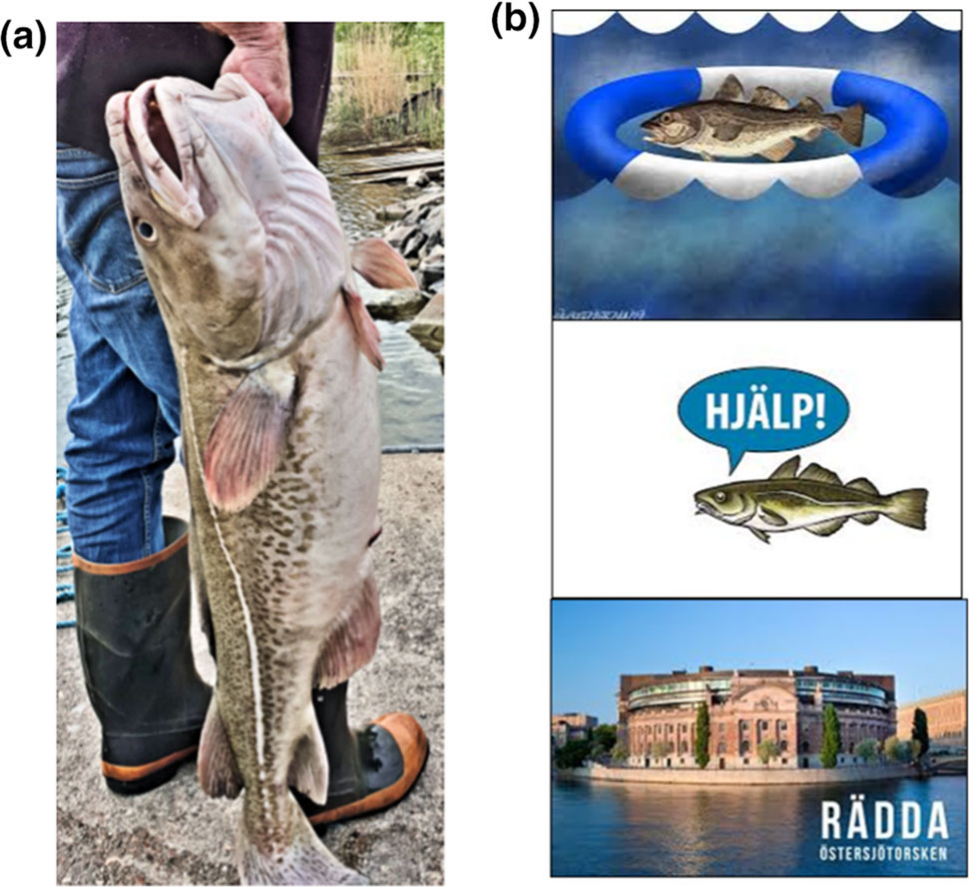
**a** A large, healthy Baltic cod. **b** Examples of images in the media and on display in public areas in Sweden during the summer of 2018, when a campaign called “Save the Baltic Cod” (“Rädda Östersjötorsken”) was undertaken to close the fishery in an attempt to halt the extirpation. The cod in the center panel is crying “Help!”. Credits: A: Photo by Y. Heimbrand. B, top panel: Wilfred Hildonen for Huvudstadsbladet hbl.fi; center panel: sportfiskarna.se; bottom panel: fisheco.se

From a social perspective, the Baltic cod is an icon (Fig. 6A), and its obvious decline in size and condition has become widely publicized by the news and other media, and has turned cod into a “poster child” for the state of the sea. Decades of popular focus on the problem of overfishing came to a head in 2018 in Sweden, when large campaigns to “Save the Baltic Cod” blanketed the airwaves and public places with imagery (Fig. 6B). This is a form of “information service” that sends a message to people about the impacted state of the system, and in this case raised alarms. The concern thus raised placed extra pressure, perhaps, on the scientists who convened the following winter to conduct a benchmark stock assessment for Baltic cod. Their comprehensive review of all the biological and fisheries data, together with the biophysical data on worsening habitat conditions, led to the scientists recommending closure of the fisheries, to allow the cod population a chance to recover (ICES 2020).

This work illustrates the impact of deoxygenation on the decrease in maximum length of Baltic Sea cod (a demographic parameter) and the subsequent effects on several aquatic ecosystem services. We show that the extent of suitable areas for cod (oxygen concentration ≥ 1 ml l^−1^) is the most important predictor when explaining the decrease of cod *L*_max_. This finding has implications for the management strategy of cod because it points to the fact that, even when reducing the fishing mortality of cod, the cod population will potentially not manage to regain a healthy size distribution unless the bottom oxygen conditions of the Baltic improve. This in turn, has had ramifications on the parts of society that base their economies on cod fishing, both commercially and recreationally, as well as having an effect on common perceptions of the health of the Baltic Sea.

## Conclusion

Deoxygenation, together with rising ocean temperatures, is predicted to lead to reduced size and hence biomass of marine fishes (Cheung et al. 2012), resulting in threats to many fisheries (Rose et al. 2019 and references therein). More studies are needed to quantify the impacts of deoxygenation on the changes in *L*_max_ of cod and other species, and the subsequent effects on ecosystem services. Nevertheless, this study provides a step towards understanding how a strong environmental driver (hypoxia) affects ecosystem services resulting from biological attributes of a key top predator. Further studies are also needed to provide additional quantification of the socioeconomic impacts of deoxygenation on the Baltic Sea ecosystem and other impacted systems, and fish such as cod will be leading indicators.

This study underlines the complex web of socio-ecological consequences emanating from a change in a key demographic parameter—maximum length of an iconic fish species—due to the environmental pressure of deoxygenation. Although the large-scale policy implication is for immediate steps to reduce greenhouse gas emissions, there is also a continuing need for policy attention to both local/regional anthropogenic drivers (nutrient management) and to fisheries management, which needs to account for the stresses induced by severe hypoxia (Rose et al. 2019). The Baltic Sea situation highlights how deoxygenation, a problem increasing in scope and severity across the globe, will require managers to “thread the needle” carefully for sustainable resource use and conservation.
